# Altered Mitochondrial Function in MASLD: Key Features and Promising Therapeutic Approaches

**DOI:** 10.3390/antiox13080906

**Published:** 2024-07-26

**Authors:** Tatjana Radosavljevic, Milica Brankovic, Janko Samardzic, Jasmina Djuretić, Dusan Vukicevic, Danijela Vucevic, Vladimir Jakovljevic

**Affiliations:** 1 Institute of Pathophysiology “Ljubodrag Buba Mihailovic”, Faculty of Medicine, University of Belgrade, 11000 Belgrade, Serbia; danijela.vucevic@med.bg.ac.rs; 2 Institute of Pharmacology, Clinical Pharmacology and Toxicology, Faculty of Medicine, University of Belgrade, 11000 Belgrade, Serbia; milicabrankovic137@yahoo.com (M.B.); jankomedico@yahoo.es (J.S.); 3 Department of Pathobiology, Faculty of Pharmacy, University of Belgrade, 11000 Belgrade, Serbia; jasmina.djuretic@pharmacy.bg.ac.rs; 4 Uniklinik Mannheim, Theodor-Kutyer-Ufer 1-3, 68167 Mannheim, Germany; vukdusan@hotmail.com; 5 Department of Physiology, Faculty of Medical Sciences, University of Kragujevac, Svetozara Markovica 69, 34000 Kragujevac, Serbia; drvladakgbg@gmail.com; 6 Center of Excellence for the Study of Redox Balance in Cardiovascular and Metabolic Disorders, University of Kragujevac, Svetozara Markovica 69, 34000 Kragujevac, Serbia; 7 Department of Human Pathology, First Moscow State Medical University I.M. Sechenov, Trubetskaya Street 8, Str. 2, 119991 Moscow, Russia

**Keywords:** mitochondria, MASLD, MASH, mitochondrial dysfunction, metabolic syndrome, oxidative stress, mitochondrial quality control

## Abstract

Metabolic dysfunction-associated steatotic liver disease (MASLD), formerly known as nonalcoholic fatty liver disease (NAFLD), encompasses a range of liver conditions from steatosis to nonalcoholic steatohepatitis (NASH). Its prevalence, especially among patients with metabolic syndrome, highlights its growing global impact. The pathogenesis of MASLD involves metabolic dysregulation, inflammation, oxidative stress, genetic factors and, notably, mitochondrial dysfunction. Recent studies underscore the critical role of mitochondrial dysfunction in MASLD’s progression. Therapeutically, enhancing mitochondrial function has gained interest, along with lifestyle changes and pharmacological interventions targeting mitochondrial processes. The FDA’s approval of resmetirom for metabolic-associated steatohepatitis (MASH) with fibrosis marks a significant step. While resmetirom represents progress, further research is essential to understand MASLD-related mitochondrial dysfunction fully. Innovative strategies like gene editing and small-molecule modulators, alongside lifestyle interventions, can potentially improve MASLD treatment. Drug repurposing and new targets will advance MASLD therapy, addressing its increasing global burden. Therefore, this review aims to provide a better understanding of the role of mitochondrial dysfunction in MASLD and identify more effective preventive and treatment strategies.

## 1. Introduction

**Figure 1:**
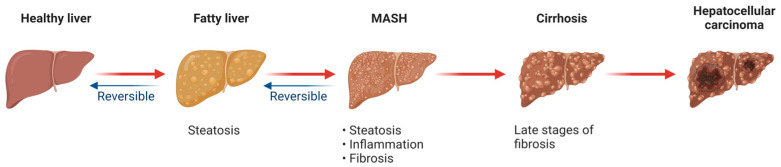
Progression of metabolic dysfunction-associated steatotic liver disease (MASLD). Created with BioRender.com. Accessed on 22 July 2024.

Metabolic dysfunction-associated steatotic liver disease (MASLD), previously recognized as nonalcoholic fatty liver disease (NAFLD), encompasses a spectrum of liver conditions ranging from steatosis to nonalcoholic steatohepatitis (NASH), which involves inflammation and potential liver damage [1]. The renaming from NAFLD to MASLD reflects a broader understanding of the disease’s underlying mechanisms and its association with metabolic dysfunction. The term NASH has also been revised to metabolic dysfunction-associated steatohepatitis (MASH) [2,3]. MASLD is commonly associated with obesity, insulin resistance, type 2 diabetes mellitus (T2DM), dyslipidemia, and metabolic syndrome [4]. It is becoming one of the most common chronic liver diseases worldwide, with the global prevalence ranging from 25% to 45%. The prevalence is shown to be notably higher among people with metabolic dysfunction [5]. As metabolic dysfunction becomes more prevalent globally, it further contributes to the rising burden of MASLD. Approximately 20–30% of MASLD patients may progress to MASH, potentially resulting in further complications such as liver cirrhosis and hepatocellular carcinoma (Figure 1) [6].

The pathogenesis of MASLD is multifactorial and involves a complex interplay of various mechanisms, including metabolic dysregulation, inflammation, oxidative stress, and genetic factors [7]. Recently, it has been suggested that mitochondrial dysfunction is strongly associated with the development and progression of MASLD [8,9,10]. Therefore, this review aims to provide a better understanding of the role of mitochondrial dysfunction in MASLD and identify more effective preventive and treatment strategies.

## 2. Mitochondrial Biology

### 2.1. Mitochondria and Oxidative Phosphorylation

Mitochondria are essential cellular organelles responsible for various critical functions, including energy production through oxidative phosphorylation (OXPHOS). OXPHOS is a metabolic pathway that occurs in the inner mitochondrial membrane and involves a sequence of enzymatic reactions that generate adenosine triphosphate (ATP), the primary energy-supplying molecule for cells [11]. During OXPHOS, electrons derived from the breakdown of nutrients are transferred through a series of protein complexes (complexes I to IV) in the mitochondrial electron transport chain (ETC). As electrons pass through these complexes, energy is released and used to pump protons (H+) across the inner mitochondrial membrane, creating an electrochemical gradient known as the proton gradient or proton motive force. The ATP synthase enzyme (complex V) is powered by the proton gradient, facilitating the phosphorylation of adenosine diphosphate (ADP) into ATP. The production of ATP is essential for numerous cellular processes, including muscle contraction, biosynthesis, and active transport [12]. Mitochondrial dysfunction, characterized by impaired OXPHOS, can lead to a range of cellular and physiological abnormalities [13].

### 2.2. Mitochondrial Oxidative Stress

Mitochondrial oxidative stress arises from an imbalance between the production of reactive oxygen species (ROS) and the ability of the cell’s antioxidant defenses to neutralize them. Mitochondria play a major role in generating ROS within cells, primarily due to the ETC activity during OXPHOS [14]. Mitochondrial oxidative stress involves a cascade of interconnected mechanisms. Initially, electron leakage from the ETC complexes can engage with molecular oxygen, resulting in the formation of superoxide radicals. Subsequently, these superoxide radicals can undergo enzymatic and non-enzymatic reactions to form other ROS, such as hydrogen peroxide (H_2_O_2_). This process is exacerbated by dysregulation of the ETC or impaired mitochondrial function, which amplifies ROS production. Factors like mitochondrial DNA (mtDNA) mutations, defects in ETC complexes, and loss of membrane potential contribute to this increased ROS generation. Additionally, mitochondria possess various antioxidant defenses to counteract ROS accumulation. Antioxidant enzymes such as superoxide dismutase (SOD), catalase, and glutathione peroxidase, along with non-enzymatic antioxidants like glutathione (GSH), play crucial roles in scavenging ROS and maintaining redox balance [14,15]. However, when the levels of ROS surpass the capabilities of these defense mechanisms, mitochondrial proteins, lipids, and DNA are subjected to oxidative damage. This damage compromises mitochondrial function and integrity, further escalating ROS production in a vicious cycle of oxidative stress. The repercussions of mitochondrial oxidative stress can have widespread effects on cellular function and contribute to the pathogenesis of various diseases. This can cause cell damage, apoptosis, inflammation, and altered signaling pathways, ultimately leading to tissue dysfunction and disease progression [14,16].

### 2.3. Mitochondria and Cell Death

Mitochondria are key regulators of apoptosis, a programmed form of cell death essential for maintaining tissue homeostasis and eliminating damaged or unwanted cells. During apoptosis, mitochondria release pro-apoptotic proteins such as cytochrome c and Smac/DIABLO into the cytoplasm, initiating a series of events that lead to cell death. This release of apoptotic factors is regulated by proteins of the Bcl-2 family, which control mitochondrial outer membrane permeabilization (MOMP). MOMP leads to mitochondrial membrane depolarization, disruption of ATP production, and activation of caspases, ultimately resulting in cell death [17]. Mitochondria also play a role in necrosis, a form of non-programmed cell death typically associated with cellular injury or stress. Mitochondrial dysfunction, oxidative stress, and calcium overload can lead to mitochondrial permeability transition (MPT), causing mitochondrial swelling, rupture of the outer membrane, and release of pro-inflammatory molecules that promote necrotic cell death [18]. Lastly, mitochondria are degraded through mitophagy, a specialized form of autophagy that selectively targets damaged or dysfunctional mitochondria. Mitophagy helps maintain mitochondrial quality control and prevents the accumulation of dysfunctional mitochondria, which can trigger apoptosis or necrosis [19]. Dysregulation of mitophagy can lead to mitochondrial dysfunction and contribute to the development of various diseases, including MASLD [20].

### 2.4. Mitochondrial Quality Control

**Figure 2:**
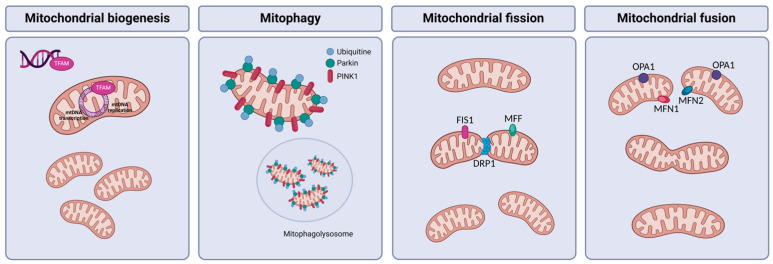
Mitochondrial quality control (MQC) mechanisms. Abbreviations: TFAM, mitochondrial transcription factor A; mtDNA, mitochondrial DNA; PINK1, PTEN-induced kinase 1; FIS1, fission protein 1; MFF, mitochondrial fission factor; DRP1, dynamin-related protein 1; OPA1, optic atrophy 1; MFN1, mitofusin 1; MFN2, mitofusin 2. Created with BioRender.com. Accessed on 22 July 2024.

Mitochondrial quality control (MQC) refers to the mechanisms by which cells maintain the health and functionality of their mitochondria. Given the critical role of mitochondria in cellular energy production, metabolism, and signaling, it is essential for cells to ensure that their mitochondria remain in a functional state. MQC involves several processes, such as biogenesis, dynamics, and mitophagy (Figure 2) [21]. Understanding the molecular mechanisms underlying impaired MQC and developing strategies to enhance or restore this process could be the key to therapeutic interventions in MASLD.

#### 2.4.1. Mitochondrial Biogenesis

Mitochondrial biogenesis is a complex process by which cells generate new mitochondria to meet their energy demands and maintain cellular homeostasis. It involves the coordinated synthesis and assembly of mitochondrial components, including proteins, lipids, and DNA [22]. Mitochondrial transcription factor A (TFAM) is a key regulator of mtDNA transcription and replication. TFAM binds to mtDNA, promoting its transcription and packaging into nucleoids, which are essential for maintaining mtDNA stability and proper mitochondrial function. The expression of TFAM is regulated by peroxisome proliferator-activated receptor-γ coactivator 1α (PGC-1α), thereby linking nuclear control of mitochondrial biogenesis to the maintenance and expression of mtDNA. Additionally, transcription factors such as nuclear respiratory factor 1 (NRF-1), nuclear respiratory factor 2 (NRF-2), and members of the estrogen-related receptor (ERR) family exert significant influence by activating promoters of mitochondrial genes [12,23]. Mitochondrial biogenesis is regulated at the post-transcriptional level as well. The translocase of the outer membrane (TOM) complex, crucial for protein import into mitochondria, can be modulated by phosphorylation by cytosolic kinases. Notably, kinases like casein kinases 1 and 2, along with protein kinase A (PRKA), have been identified as regulators that can either stimulate or inhibit protein import into mitochondria, thus impacting mitochondrial biogenesis [24].

#### 2.4.2. Mitochondrial Dynamics

Mitochondria exhibit remarkable dynamism, continuously altering their shape through fusion and fission processes. The balance between fusion and fission is regulated by various proteins encoded by nuclear genes [25]. Beyond modulating mitochondrial morphology, these dynamic processes involve different aspects of mitochondrial dynamics, including size, quantity, distribution, and intracellular transport [13].

##### Mitochondrial Fusion

Mitochondrial fusion is a dynamic process through which individual mitochondria within a cell merge their membranes and contents to form a larger, interconnected network. This fusion process is mediated by specific proteins and is essential for maintaining mitochondrial function. Key proteins involved in mitochondrial fusion include mitofusin 1 (MFN1), mitofusin 2 (MFN2), and optic atrophy 1 (OPA1) [26]. MFN1 and MFN2 are located in the outer mitochondrial membrane and facilitate the tethering and fusion of adjacent mitochondria, while OPA1 is found in the inner mitochondrial membrane and regulates the fusion of the inner mitochondrial membranes [27]. Through mitochondrial fusion, cells can exchange mitochondrial contents, including mtDNA and its encoded proteins, and ensure the proper distribution of healthy mitochondrial components.

##### Mitochondrial Fission

Mitochondrial fission is the process by which a single mitochondrion divides into two or more smaller mitochondria. This dynamic process is essential for maintaining mitochondrial quality control, distribution, and turnover within the cell. Mitochondrial fission is primarily regulated by the activity of dynamin-related protein 1 (DRP1), a GTPase that assembles into spirals around the mitochondrion at constriction sites. DRP1’s recruitment to mitochondria is facilitated by various adaptor proteins, including mitochondrial fission factor (MFF), mitochondrial dynamics proteins of 49 and 51 kDa (MiD49 and MiD51, respectively), and fission protein 1 (FIS1). Once recruited, DRP1 mediates the constriction of the mitochondrial outer membrane, leading to mitochondrial division. This process is tightly regulated by post-translational modifications, including phosphorylation, SUMOylation, and ubiquitination, as well as by interactions with other mitochondrial and cytosolic proteins [28]. Through mitochondrial fission, cells can dynamically regulate mitochondrial morphology and distribution, adapt to changing metabolic demands, and selectively eliminate damaged mitochondria through mitophagy.

#### 2.4.3. Mitophagy

As previously mentioned, mitophagy is a specialized form of autophagy that selectively targets damaged or dysfunctional mitochondria. This process plays a crucial role in maintaining mitochondrial quality control and cellular homeostasis by removing damaged mitochondria and preventing the accumulation of harmful mitochondrial components. Mitophagy is typically initiated in response to cellular stress, such as oxidative stress, nutrient deprivation, or mitochondrial dysfunction [29]. Key regulators of mitophagy include PTEN-induced kinase 1 (PINK1) and Parkin, which are involved in identifying damaged mitochondria and tagging them for degradation. PINK1 accumulates on the outer membrane of dysfunctional mitochondria, where it phosphorylates ubiquitin and recruits Parkin to ubiquitinate mitochondrial proteins. This ubiquitination serves as a signal for the autophagic machinery to engulf the damaged mitochondria and deliver them to lysosomes for degradation. Other proteins that are important for the regulation of mitophagy include Bcl-2/adenovirus E1B 19-kDa protein-interacting protein 3 (BNIP3), FUN14 domain-containing protein 1 (FUNDC1), mitophagy protein Atg32 (ATG32), optineurin (OPTN), and Bcl-2/adenovirus E1B 19-kDa protein-interacting protein 3-like (NIX/BNIP3L) [30]. Through mitophagy, cells can selectively remove dysfunctional mitochondria, thereby maintaining mitochondrial quality and preventing the release of harmful molecules that could trigger cell death or contribute to disease progression.

## 3. Mitochondrial Dysfunction and MASLD

**Figure 3:**
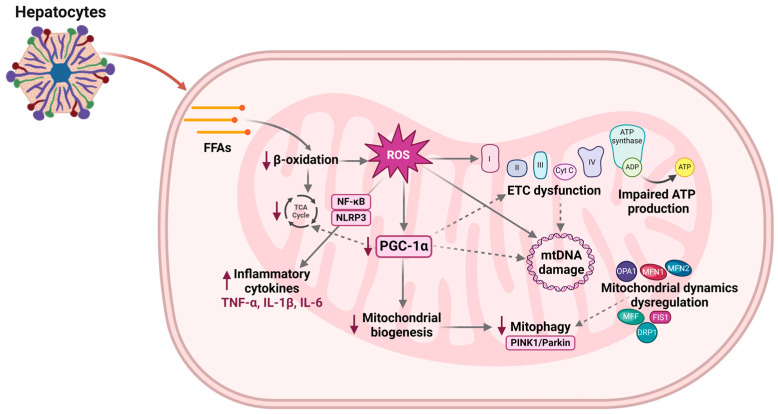
Mechanisms of mitochondrial dysfunction in MASLD. Abbreviations: FFAs, free fatty acids; ROS, reactive oxygen species; ETC, electron transport chain; Cyt C, cytochrome c; mtDNA, mitochondrial DNA; PGC-1α, peroxisome proliferator-activated receptor-γ coactivator 1α; NF-κB, nuclear factor-kappa B; NLRP3, nucleotide-binding oligomerization domain-like receptor 3; TNF-α, tumor necrosis factor-alpha; IL-1β, interleukin-1 beta; IL-6, interleukin-6; PINK1, PTEN-induced kinase 1; FIS1, fission protein 1; MFF, mitochondrial fission factor; DRP1, dynamin-related protein 1; OPA1, optic atrophy 1; MFN1, mitofusin 1; MFN2, mitofusin 2. Created with BioRender.com. Accessed on 23 July 2024.

Oxidative stress compromises mitochondrial function, leading to impaired ATP production, ETC dysfunction, and mtDNA damage (Figure 3) [14]. Dysfunctional mitochondria further contribute to ROS generation, including superoxide radicals and H_2_O_2_, as byproducts of ETC activity. Studies have shown that patients with MASH exhibit increased levels of ROS and ROS-induced mtDNA damage [31,32,33]. Elevated ROS levels contribute to oxidative stress, causing cellular damage, lipid peroxidation, and inflammation in the liver, exacerbating MASLD’s pathology. The onset of this damaging cycle of ROS is thought to be triggered by the accumulation of long-chain free fatty acids (FFAs) in hepatocytes. ROS can originate from various sources within mitochondria. It has been shown that superoxide generation primarily occurs at the flavin mononucleotide group of complex I through a process known as reverse electron transfer. ROS can also be generated by apoptosis-inducing factor (AIF), which exhibits NADH oxidase activity [34]. Prolonged stimulation of mitochondrial activity under conditions of lipid overload may cause excessive electron leakage, mainly due to the upregulation and activity of uncoupling protein-2 (UCP2) [35]. Moreover, ROS may disrupt mitochondrial permeability transition pores (MPTPs), causing the leakage of mtDNA into the cytoplasm [36]. This leakage can activate various cellular receptors, including Toll-like receptor 9 (TLR-9), triggering their subsequent signaling pathways [34]. ROS can modulate inflammatory signaling pathways, including nuclear factor-kappa B (NF-κB) and nucleotide-binding oligomerization domain-like receptor 3 (NLRP3) inflammasome signaling pathway, leading to increased production of inflammatory cytokines such as interleukin-1 beta (IL-1β), interleukin-6 (IL-6), and tumor necrosis factor-alpha (TNF-α) [37]. TNF-α intensifies oxidative damage and inflammation while also activating mitogen-activated protein kinases (MAPKs). This activation leads to the production of ROS, particularly superoxide (O_2_^•−^). The resulting oxidative stress further damages cellular components and induces the production of more TNF-α, creating a vicious cycle of inflammation and oxidative damage. Additionally, pro-inflammatory cytokines activate Kupffer cells, which further amplify the inflammatory response and exacerbate liver injury in MASLD [38].

Dysregulation of proteins involved in mitochondrial fusion (MFN1, MFN2, and OPA1) may contribute to aberrant mitochondrial morphology and impaired function in MASLD. Various studies have shown that a high-fat diet decreases the expression of MFN1, MFN2, and OPA1 [39,40]. Reduced levels of MFN2 have also been observed in liver biopsies from patients with MASH and experimental models of steatosis or NASH [41]. Furthermore, studies have demonstrated that elevated glucose levels can lead to mitochondrial fragmentation and impaired cellular function by modulation of OPA1 [42,43]. Contrary to previous findings, overexpression of OPA1 has shown beneficial effects in various experimental models [44,45]. Additionally, recent studies found that OPA1 depletion may prevent steatosis and exert protective effects in MASLD/MASH [46,47,48].

As previously mentioned, key regulators of mitochondrial fission include DRP1, MFF, MiD49 and MiD51, and FIS1. The altered expression or activity of these proteins can disrupt the balance between fission and fusion dynamics, leading to mitochondrial fragmentation and dysfunction. A recent study discovered that the expression levels of fission proteins, including DRP1 and FIS1, were increased, while there was a significant decrease in the expression of fusion proteins, such as MFN2 and OPA1, in the group of mice fed with a high-fat diet [49]. Moreover, research has shown that the expression of the dynamin 1-like (DNM1L) protein, the human homolog of DRP1, in adipose tissue is associated with obesity and insulin resistance, indicating a potential role for mitochondrial fission in metabolic disorders [50]. In a murine model of MASLD, reduced mitochondrial fission has been associated with the amelioration of hepatic steatosis [51]. This leads to the maintenance of mitochondrial integrity and function, thereby preserving lipid metabolism and reducing the accumulation of triglycerides in the liver. It also attenuates oxidative stress and inflammation, key drivers of MASLD’s progression. Moreover, increased expression of isocitrate dehydrogenase 2 (IDH2) suppresses ROS production and reduces the levels of DRP1 and FIS1 [40], suggesting a potential therapeutic target for MASLD.

The signaling pathways that regulate mitophagy, such as those involving PINK1 and Parkin, may be dysregulated in MASLD. Reduced activation of these pathways can impair the selective targeting of damaged mitochondria for degradation. Zhou et al. demonstrated a close association between MASLD and impaired Parkin-related mitophagy due to macrophage-stimulating 1 (MST1) upregulation [52]. A recent study has revealed that mitophagy dysfunction manifests early in MASLD. Additionally, this study found a correlation between reduced Parkin levels and accelerated disease progression [53]. Furthermore, the targeted deletion of lysocardiolipin acyltransferase 1 (ALCAT1) has been shown to reverse mitophagy arrest and mitigate mitochondrial dysfunction in MASLD. Depletion of BNIP3 resulted in increased lipid synthesis and disruption of mitochondrial membrane integrity [54]. Dysregulation of mitochondrial dynamics, including aberrant mitochondrial fusion and fission processes, can also affect mitophagy [55]. Fragmented mitochondria resulting from excessive fission may be less efficiently targeted for degradation. Accumulation of dysfunctional mitochondria further exacerbates ROS production and cellular stress, contributing to sustained inflammation. Damaged mitochondria can also release mtDNA into the cytoplasm, which is recognized by the immune system as a damage-associated molecular pattern (DAMP). This recognition further stimulates inflammatory pathways, amplifying the inflammatory response. Inflammatory cytokines, including interleukin-1 alpha (IL-1α), IL-1β, and interleukin-18 (IL-18), may interfere with mitophagy signaling pathways, exacerbating mitochondrial dysfunction [56].

## 4. Potential Therapeutic Approaches for MASLD

**Figure 4:**
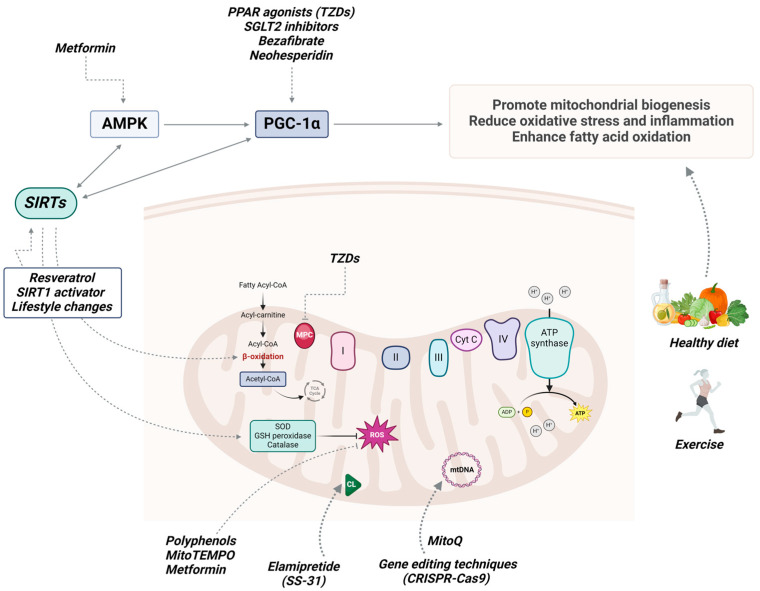
Mitochondria-targeted agents and lifestyle changes in MASLD. Abbreviations: AMPK, AMP-activated protein kinase; PGC-1α, peroxisome proliferator-activated receptor-γ coactivator 1α; SIRTs, sirtuins; PPAR, peroxisome proliferator-activated receptor; TZDs, thiazolidinediones; SGLT2, sodium–glucose co-transporter 2; MitoQ, Mito-quinone; MPC, mitochondrial pyruvate carrier; Cyt C, cytochrome c; mtDNA, mitochondrial DNA; CL, cardiolipin; ROS, reactive oxygen species; SOD, superoxide dismutase; GSH, glutathione. Created with BioRender.com. Accessed on 22 July 2024.

There is a growing interest in exploring the therapeutic potential of enhancing mitochondrial function in MASLD (Table 1). Various strategies have been proposed in this regard, including combinations of lifestyle modifications and pharmacological therapy, each aimed at distinct objectives in addressing the complex pathology of MASLD (Figure 4) [57]. Until recently, there were no medications specifically approved for the treatment of MASLD. However, in March 2024, the FDA approved the drug resmetirom (Rezdiffra) for the treatment of MASH with moderate-to-advanced fibrosis [58]. Resmetirom, a selective thyroid hormone receptor-β (THR-β) agonist, demonstrated a favorable side-effect profile, significant improvements in fibrosis, and a reduction in MASLD progression [59]. By activating THR-β, a nuclear hormone receptor, resmetirom modulates various genes that promote mitochondrial biogenesis, mitophagy, and β-oxidation in hepatocytes [59,60]. Resmetirom is now a recommended treatment for adult patients with MASH, along with lifestyle modifications [58].

Lifestyle modifications, including exercise and a healthy diet, have demonstrated efficacy in improving mitochondrial function among patients with MASLD [61]. Regular physical activity has been associated with improvements in various aspects of MASLD’s pathophysiology. Exercise has been shown to reduce fat accumulation in the liver by enhancing fatty acid oxidation and inhibiting lipogenesis, primarily through the activation of the AMP-activated protein kinase (AMPK) pathway [62]. Moreover, the beneficial effects of physical activity on reducing chronic inflammation and oxidative stress are well established [63]. Exercise can reduce the production of pro-inflammatory cytokines such as TNF-α and IL-6 while promoting the release of anti-inflammatory cytokines such as interleukin-10 (IL-10) [64]. A recent study demonstrated that exercise-induced irisin, a PGC1-α-dependent myokine, has anti-inflammatory effects and improves MASLD by competitively binding with myeloid differentiation factor 2 (MD2) [65]. Additionally, engaging in regular exercise increases the production of endogenous antioxidants such as SOD, GSH peroxidase, and catalase [66]. While the precise effect of exercise on mitochondrial quality in MASLD remains to be fully elucidated, a recent experimental study suggested that exercise improves mitochondrial function/morphology and enables mitophagy [67]. These findings emphasize the potential of exercise as a strategy for targeting mitochondria and treating MASLD.

The impact of a nutritious diet on mitochondrial function is also an area of ongoing research. Opting for nutrient-dense foods such as fruits, vegetables, whole grains, and lean proteins provides essential vitamins, minerals, and antioxidants that are important for mitochondrial health [68]. Conversely, diets rich in processed foods, sugars, and saturated fats can cause inflammation, insulin resistance, and oxidative stress, thereby exacerbating mitochondrial dysfunction [69]. The Mediterranean diet (MedDiet), abundant in extra-virgin olive oil, omega-3 fatty acids, fruits, and polyphenol-rich plants and vegetables, ameliorates hepatic steatosis in patients with MASLD [70]. Moreover, numerous studies have demonstrated the benefits of the MedDiet on mitochondrial metabolism and biogenesis. Polyphenols possess antioxidant properties that help to scavenge ROS, thereby reducing oxidative stress and inflammation [71,72]. Additionally, polyphenols can modulate mitochondrial biogenesis, as well as regulating mitochondrial membrane potential and mitochondrial enzyme activity [73]. Resveratrol has shown antioxidative and anti-inflammatory properties by activating sirtuin 1 (SIRT1) and triggering the AMPK pathway [72]. Additionally, resveratrol acts as a PGC-1α activator alongside compounds like bezafibrate, thereby promoting mitochondrial biogenesis [74,75]. Similarly, neohesperidin has been shown to enhance PGC-1α-mediated mitochondrial biogenesis and reduce hepatic steatosis [76]. Hydroxytyrosol, a polyphenol from olive oil, has demonstrated the potential to improve mitochondrial function and alleviate MASLD. This effect may be mediated by its ability to activate mitophagy through the AMPK/PINK1 pathway [77]. Berberine, an isoquinolone alkaloid, enhances the activity of sirtuin 3 (SIRT3) and improves OXPHOS [78]. Various studies suggest that berberine holds promise as a therapeutic approach for MASLD by modulating inflammatory signaling pathways and reducing oxidative stress [79,80,81]. Genistein, an isoflavone, has also gained attention for its potential role in treating MASLD. Studies suggest that genistein possesses anti-inflammatory, antioxidant, and lipid-lowering properties. Additionally, genistein may exert protective effects on hepatocytes through its ability to regulate insulin sensitivity and improve mitochondrial function [82,83]. Flavonoids, such as silymarin and silybin-phospholipid, have recently shown hepatoprotective effects, primarily by reducing oxidative stress and preventing mitochondrial dysfunction [84,85]. Among their numerous functions, silymarin helps maintain the integrity of mitochondrial membranes, while silybin-phospholipid regulates mitochondrial energy metabolism [74]. In a recent experimental study, avocado oil has been shown to decrease inflammation, improve mitochondrial function, and reduce oxidative stress [86]. Although the roles of different natural compounds are still being investigated, emerging evidence strongly suggests that targeting oxidative stress represents a promising strategy for MASLD.

Mitochondria-targeted agents, including Mito-quinone (MitoQ), MitoTEMPO, and elamipretide (SS-31), represent innovative approaches aimed at directly targeting mitochondrial dysfunction [87]. These agents offer potential therapeutic benefits for a variety of disorders associated with impaired mitochondrial function, including MASLD. MitoQ acts as an antioxidant, scavenging harmful ROS and reducing oxidative stress. By reducing oxidative damage within the mitochondria, MitoQ helps to preserve mitochondrial function and integrity [88]. Williamson et al. have recently demonstrated that MitoQ supplementation has a protective effect on mtDNA [89]. Furthermore, numerous studies have shown that the combination of MitoQ with other agents improves mitochondrial dysfunction and reduces oxidative stress [90,91,92]. Elamipretide (SS-31) improves mitochondrial function by targeting cardiolipin, a phospholipid in the inner mitochondrial membrane. By interacting with cardiolipin, elamipretide stabilizes mitochondrial membranes, improves ETC activity, and reduces oxidative stress [93]. The beneficial effects of elamipretide have been demonstrated in numerous studies and various conditions [93,94,95]. Another novel compound, MitoTEMPO, neutralizes ROS and helps maintain mitochondrial function and integrity [96,97,98]. Although the beneficial effects of these drugs have been demonstrated in various diseases, their precise role in treating MASLD is still being investigated.

PPAR agonists work by activating peroxisome proliferator-activated receptors (PPARs), which are nuclear receptors that play a key role in regulating various metabolic processes, including lipid metabolism, inflammation, and insulin sensitivity. There are three subtypes of the PPAR receptor family: PPAR-α, PPAR-δ, and PPAR-γ [99]. PPAR-α regulates genes that are crucial for peroxisomal and mitochondrial β-oxidation [100]. PPAR-δ plays an important role in enhancing mitochondrial function and promoting mitochondrial biogenesis [101]. PPAR-γ is also involved in mitochondrial biogenesis by activating transcription factors such as PGC-1α [102]. Elafibranor, a dual PPAR-α/δ agonist, is currently pending approval for the therapy of primary biliary cholangitis. Results from the ELATIVE Phase III trial demonstrated its efficacy and safety [103]. While several studies have shown promising results suggesting that elafibranor improves steatosis, inflammation, and fibrogenesis in MASLD/MASH [104,105,106], the results from the REVOLVE-IT Phase III trial did not demonstrate the efficacy of elafibranor in comparison to the placebo [107]. Thiazolidinediones (TZDs), as PPAR-γ agonists, are primarily indicated for the treatment of T2DM due to their ability to increase insulin sensitivity [108]. However, their potential use in MAFLD/MASH has also been explored. These drugs have shown promise in preclinical and clinical studies for their ability to improve insulin sensitivity and reduce hepatic steatosis, inflammation, and fibrosis [109,110,111]. Various experimental studies have shown that TZDs, such as pioglitazone, may promote mitochondrial biogenesis and enhance mitochondrial function [112,113,114]. Their mechanism of action involves the induction of PGC-1α, which regulates genes involved in lipid metabolism, adipogenesis, insulin sensitivity, and inflammation. Moreover, TZDs inhibit the mitochondrial pyruvate carrier (MPC), which is responsible for transporting pyruvate across the mitochondrial inner membrane, reducing the flow of pyruvate into mitochondrial metabolic pathways [115]. Azemiglitazone potassium (MSDC-0602K) is a new medication classified as a PPAR-γ-sparing thiazolidinedione (Ps-TZD). It represents a novel approach to insulin sensitization by retaining the beneficial effects of TZDs while targeting MPC [116]. By sparing PPAR-γ activation, drugs like MSDC-0602K may offer potential benefits in terms of metabolic control while reducing the risk of side effects commonly associated with conventional TZDs [117]. MSDC-0602K is currently undergoing Phase III clinical trials involving patients with pre-T2DM or diagnosed T2DM who also exhibit signs of MASLD/MASH [118]. Furthermore, studies have highlighted the efficacy of saroglitazar, another dual PPAR-α/γ agonist, in reducing steatosis, alongside favorable improvements in various metabolic parameters [119,120,121]. Lanifibranor, the first pan-PPAR agonist targeting three distinct PPAR isotypes, is currently undergoing Phase III clinical trials involving patients diagnosed with MASH and fibrosis [122]. The Phase IIb trial results showed significant improvements in hepatic steatosis, inflammation, and fibrosis [123]. These trials mark a significant advancement in liver disease research, as they address the critical need for effective treatments for MASLD and MASH.

Sodium-glucose co-transporter 2 (SGLT2) inhibitors, such as empagliflozin and dapagliflozin, are a class of medications used in the treatment of T2DM, heart failure, and chronic kidney disease [124]. Numerous studies have shown their beneficial effects in improving mitochondrial function among a range of health conditions [125,126,127,128,129,130]. Some proposed mechanisms for modulating mitochondrial function include upregulating transcription factors like PGC-1α and TFAM, regulating mitophagy through FIS1, MFN1, MFN2, and OPA1, and impacting various cellular pathways [126,131,132]. Evidence suggests that SGLT2 inhibitors decrease the production of ROS and help maintain ion homeostasis [133,134]. In an experimental model of T2DM induced by a high-fat diet combined with streptozotocin, chronic administration of dapagliflozin has shown promising effects on mitochondrial morphology and OXPHOS. Specifically, dapagliflozin normalized the mitochondrial size in hepatocytes, increased the mitochondrial DNA copy number, and upregulated the expression of genes involved in mitochondrial dynamics, such as PGC-1α, MFN2, and DRP1 [125]. Additionally, low concentrations of dapagliflozin may protect mitochondria by reducing H_2_O_2_ production, thereby mitigating oxidative stress. However, this effect was not observed with an increase in the dose [135]. Another SGLT-2 inhibitor, ipragliflozin, demonstrated antioxidative and anti-inflammatory properties through the modulation of distinct pathways, potentially improving the progression of MASH [136,137]. A recent study indicated that SGLT2 inhibitors are the preferred choice among other antidiabetic drugs for patients with MASLD [138]. Moreover, positive outcomes were observed in the Phase II LEGEND study, which investigated the combination of lanifibranor with empagliflozin in patients diagnosed with non-cirrhotic MASH and T2DM. Notably, patients treated with lanifibranor, alone or in combination with empagliflozin, had significant reductions in hepatic steatosis and fibrosis [139]. Despite these promising findings and the presence of strong evidence suggesting positive effects of SGLT2 inhibitors in MASLD [138,140,141], further investigation is needed to better understand their role in modulating mitochondrial function.

Metformin has gained attention for its potential therapeutic role in MASLD, beyond its conventional use in T2DM [142]. Among its various established mechanisms, metformin activates AMPK, a cellular energy sensor that regulates multiple metabolic processes. Activation of AMPK leads to increased mitochondrial biogenesis, improved mitochondrial function, and enhanced fatty acid oxidation [143]. Metformin also exhibits antioxidant properties by modulating mitochondrial respiratory chain complex I [142,144]. In an animal model of palmitate-induced hepatic cell death, metformin reduced cellular ROS production while simultaneously increasing the production of SOD [145]. These effects are believed to occur through an AMPK-independent mechanism, suggesting alternative mechanisms at play. Experimental studies have revealed further possible mechanisms of metformin’s action, including the inhibition of mitochondrial glycerol-3-phosphate dehydrogenase (mGPDH) and mitochondrial respiratory chain complex IV [146,147]. Additionally, metformin has demonstrated positive effects on mitophagy and biogenesis, as evidenced by increased levels of the mitophagy markers PINK1 and Parkin, alongside the mitochondrial biogenesis marker PGC1α [148].

Sirtuins, particularly SIRT1, SIRT3, and SIRT6, play an important role in regulating mitochondrial quality control, making them promising targets for the treatment of mitochondrial dysfunction [149]. SIRT1 activation increases the expression of genes involved in mitochondrial biogenesis, including the upregulation of PGC-1α, a key regulator in this process. In an animal model of MASLD, decreased levels of SIRT1 and PGC-1α were observed [150]. Activation of SIRT3 increases the expression of proteins involved in mitochondrial fusion, such as OPA1, MFN2, and DRP1. Moreover, SIRT3 enhances the enzymatic activity of SOD2, leading to a reduction in oxidative stress [151]. Similarly, SIRT6 activation can improve mitochondrial function and reduce oxidative stress [152]. As mentioned earlier, lifestyle modifications such as exercise and calorie restriction, along with compounds like resveratrol, have demonstrated the ability to activate both SIRT1 and SIRT3. In recent years, several small-molecule modulators have been developed, and they are currently undergoing clinical trials. Among them, SRT2104 (SIRT1 activator) stands out as one of the most promising candidates [153]. Other members of the SIRT family have also demonstrated protective effects in MASLD [154,155]. However, additional research is necessary to fully understand their therapeutic potential.

Among the promising strategies to alleviate mitochondrial dysfunction is gene therapy. Gene therapy approaches aim to correct genetic mutations or defects affecting mitochondrial function. This may involve gene editing techniques such as CRISPR-Cas9 and targeted gene therapies. Numerous candidate genes associated with the progression and development of MASLD/MASH have been identified [156]. While gene therapy for MASLD is not yet available, there has been substantial advancement in treating Leber’s hereditary optic neuropathy (LHON). The Phase III clinical trials evaluating the efficacy and safety of lenadogene nolparvovec have demonstrated positive outcomes [157]. This progress represents a significant step forward in utilizing gene therapy for mitochondrial disorders, paving the way for developing treatments for various conditions associated with mitochondrial dysfunction, including MASLD.

## 5. Conclusions

Strong evidence of an association between MASLD and mitochondrial dysfunction underscores the urgent need for effective therapeutic interventions. The dysregulation of mitochondrial processes contributes significantly to MASLD’s pathogenesis. Targeting mitochondrial dysfunction through innovative therapies, such as gene editing techniques and small-molecule modulators, along with lifestyle interventions, holds promise in mitigating MASLD’s progression. Additionally, repurposing drugs and identifying new targets are crucial aspects that can enhance the efficacy of MASLD therapy. However, further research is essential for a better understanding of the specific mechanisms underlying MASLD-related mitochondrial dysfunction, and for the development of targeted therapies.

**Table 1:** List of potential mitochondria-enhancing therapies for MASLD.

Category	Name	Function	References
PPAR agonists	Elafibranor Thiazolidinediones Pioglitazone Azemiglitazone potassium (MSDC-0602K)	Improves steatosis, inflammation, and fibrosis Potential effects on mitochondria Promotes mitochondrial biogenesis Inhibits the mitochondrial pyruvate carrier (MPC)	[104,105,106] [112,113,114] [115,116,117]
SGLT2 inhibitors	Empagliflozin Ipragliflozin Dapagliflozin	Decreases ROS production Up-regulates transcription factors (PGC-1α and TFAM), regulate mitophagy Normalizes mitochondrial size in hepatocytes Increases mtDNA copy number	[133,134] [126,131] [125,135]
Biguanides	Metformin	Increases β-oxidation and mitochondrial biogenesis Modulates mitochondrial respiratory chain complexes I and IV	[143] [144,146]
Sirtuins (small molecule modulators)	SRT2104 (SIRT1 activator)	Increases the expression of genes involved in mitochondrial biogenesis	[153]
Mitochondria-targeted agents	MitoQ MitoTEMPO Elamipretide (SS-31)	Reduces oxidative stress Protective effect on mtDNA Reduces oxidative stress Interacts with cardiolipin, which stabilizes mitochondrial membranes, improves ETC activity and reduces oxidative stress	[88] [89] [96,97,98] [93]
Gene therapy		Corrects genetic mutations or defects affecting mitochondrial function	[156]
